# CaSK23, a Putative GSK3/SHAGGY-Like Kinase of *Capsicum annuum*, Acts as a Negative Regulator of Pepper’s Response to *Ralstonia solanacearum* Attack

**DOI:** 10.3390/ijms19092698

**Published:** 2018-09-11

**Authors:** Ailian Qiu, Ji Wu, Yufen Lei, Yiting Cai, Song Wang, Zhiqin Liu, Deyi Guan, Shuilin He

**Affiliations:** 1 College of Life Science, Fujian Agriculture and Forestry University, Fuzhou 350002, China; ailian808@163.com (A.Q.); wuji7166@sina.com (J.W.); 2 Key Laboratory of Crop Genetics and Breeding and Comprehensive Utilization, Ministry of Education/FAFU, Fuzhou 350002, China; fern382@163.com (Y.L.); yuanwanggood@163.com (Y.C.); wangsong1699@126.com (S.W.); lzqfujian@126.com (Z.L.); zwxy069@163.com (D.G.)

**Keywords:** Pepper, *CaSK23*, VIGS, overexpression, immunity

## Abstract

GSK3-like kinases have been mainly implicated in the brassinosteroids (BR) pathway and, therefore, in plant growth, development, and responses to abiotic stresses; however, their roles in plant immunity remain poorly understood. Herein, we present evidence that CaSK23, a putative GSK3/SHAGGY-like kinase in pepper, acts as a negative regulator in pepper’s response to *Ralstonia solanacearum* (*R. solanacearum*) inoculation (RSI). Data from quantitative RT-PCR (qRT-PCR) showed that the constitutively-expressed *CaSK23* in pepper leaves was down-regulated by RSI, as well as by exogenously-applied salicylic acid (SA) or methyl jasomonate (MeJA). Silencing of *CaSK23* by virus-induced gene silencing (VIGS) decreased the susceptibility of pepper plants to RSI, coupled with up-regulation of the tested genes encoding SA-, JA-, and ethylene (ET)-dependent pathogenesis-related (PR) proteins. In contrast, ectopic overexpression (OE) of *CaSK23* conferred a compromised resistance of tobacco plants to RSI, accompanied by down-regulation of the tested immunity-associated SA-, JA-, and ET-dependent PR genes. In addition, transient overexpression of *CaSK23* in pepper plants consistently led to down-regulation of the tested SA-, JA-, and ET-dependent PR genes. We speculate that CaSK23 acts as a negative regulator in pepper immunity and its constitutive expression represses pepper immunity in the absence of pathogens. On the other hand, its decreased expression derepresses immunity when pepper plants are attacked by pathogens.

## 1. Introduction

During their life cycles, plants are frequently exposed to various stresses, such as pathogens, in their habitats. To survive, plants constantly remodel their growth, development, and defense reactions in a coordinated manner by transcriptional, metabolic, and physiological reprogramming. For example, plants prioritize defense over growth in the presence of stress [1,2], and then turn off defense reactions in the absence or resolution of stress [3]. This adaptive behavior requires the accurate perception and transformation of stress signals into appropriate defense outputs by signaling networks and transcriptional cascades. Identified signaling cascades include signals mediated by Ca^2+^ [4,5,6,7,8], GTPases [9,10], Reactive oxygen species (ROS) [8,11,12], phytohormones such as salicylic acid (SA) [13,14,15], jasmonic acid (JA) [16,17], ethylene (ET) [18], ABA [19], brassinosteroid (BR) [20], cytokinin (CK), and gibberellin (GA) [6,21,22,23], as well as by various kinases, phosphatases [24,25,26], or transcription factors [27,28,29]. These cascades might act as positive or negative regulators to fine-tune plant defense responses, leading to appropriate immune outputs. However, the majority of components in these signaling networks remain to be identified.

GSK3 (Glycogen synthase kinase 3)-like kinases, also known as SHAGGY-like kinases, were originally identified in animals [30], and were then also found in plants [31]. GSK3 proteins are encoded by multiple gene families in plants, with 10 members in *Arabidopsis* [32], and GSK family members can be classified into four subgroups [33]. Early studies focused on the roles of GSK3-like kinases in BR signaling [33,34,35,36,37,38]. For example, the crucial component BIN2, one of the three members in group II of GSK3-like kinases, is a negative regulator in the BR signaling pathway [31,38,39,40,41]. In addition to their role in BR signaling, GSK3 kinases have also been implicated in processes such as carbohydrate metabolism [42], cell growth, root and stomatal cell development, flower development, xylem differentiation, light response, abiotic stress responses [37,43,44], and plant immunity [45,46,47,48,49]. GSK3 kinases also influence the crosstalk among auxin, JA, abscisic acid, and BR pathways [34,41,50,51,52,53]. As the information about GSK3 kinases comes from the model plant *Arabidopsis*, the roles of GSK3 family members in other non-model plants, especially in immunity, remain poorly understood.

The productivity and quality of pepper plants (*Capsicum annuum*), a crop of worldwide agricultural importance, are frequently decreased by diseases such as Phytophthora blight and bacterial wilt *R. solanacearum* is a devastating soil-borne bacterium that causes wilting disease in over 200 economically-important plant species, including pepper and tobacco, and the bacterial wilting caused by *Ralstonia solanacearum* is one of the most influential diseases affecting pepper production worldwide. An analysis of pepper’s transcriptional response to *R. solanacearum* inoculation (RSI) revealed a putative GSK, named CaSK23, which was down-regulated by RSI. We studied its role in pepper immunity against RSI, and our data indicated that CaSK23 acts as a negative regulator in pepper resistance to *R. solanacearum* attack.

## 2. Results

### 2.1. Cloning and Sequence Analysis of CaSK23

A positive cDNA clone was acquired based on the cDNA-AFLP assay on differentially-expressed pepper leaf genes after exogenous application of SA, as well as the PCR-based cDNA library screening. Its deduced amino acid sequence harbors a conserved Ser/Thr kinase domain, in which the highly conserved arginine 144, arginine 229, and lysine 254 constitute a phosphate binding pocket (Figure S1a) [54]. The predicted molecular weight and theoretical isoelectric point of the deduced protein were 50,486.0 and 8.0 Da, respectively. Because homology searching revealed that the deduced amino acid sequence shares the highest sequence similarities to AtSK23 (77.8%) among all of the clade II GSK kinases in *Arabidopsis*, including AtSK21/BIN2 (77.4%), AtSK22/AtGSK1 (73.8%), and AtSKetha (72.7%) (Figure S1b), it was named CaSK23 (SK of *Capsicum annuum*). In addition, CaSK23 shares 86.1%, 70.9%, 73.6%, and 65.8% sequence similarities with PSK8, PSK9 (*Petunia hybrida*), SK1-A (*Triticumaestivum*), and SK2 (*Physcomitrella patens*), respectively. To our knowledge, this is the first GSK to be characterized in pepper plants.

### 2.2. Transcriptional Expression of CaSK23 in Pepper Organs and Leaves after R. solanacearum or Exogenoushormones

**Figure 1:**
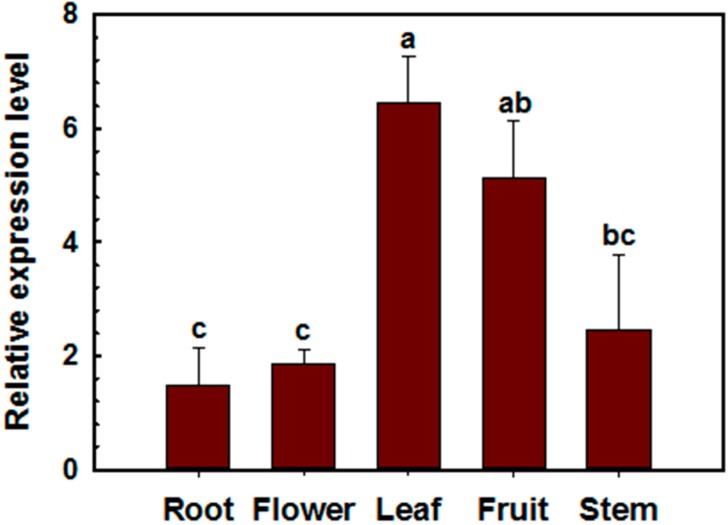
Quantitative RT-PCR (qRT-PCR) analysis of *CaSK23* in different organs of three-month-old pepper plants. Root transcript levels were used as the reference, which was set as ‘1’. Each value is the average of three replicate experiments ± SD. Different lowercase letters indicate statistically significant differences (Fisher’s protected LSD test; *p* < 0.05).

**Figure 2:**
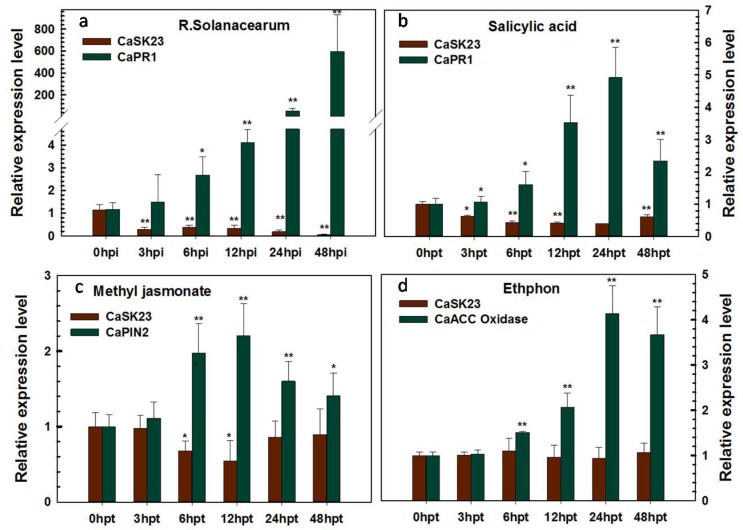
qRT-PCR analysis of relative *CaSK23* transcript levels in pepper plants challenged with *Ralstonia solanacearum* (*R. solanacearum*) inoculation (RSI) and an exogenous application of phytohormones. (**a**) Transcription levels of *CaSK23* in the RSI pepper plants. Pepper plants were inoculated with 10 μL of the highly virulent *R. solanacearum* strain FJC100301 suspension (OD_600_ = 0.8) (using 10 mM MgCl_2_ as mock treatment) in the lateral vein of the third leaves from the top, and the fourth leaves were harvested at indicated time points for RNA extraction and qPCR. (**b**–**d**) Relative transcript levels of *CaSK23* in pepper leaves at various time points after spraying with 5 mM salicylic acid, 100 µM methyl jasmonate, and 10 m Methephon or mock. (**a**–**d**) Relative transcript levels of *CaSK23* in pepper leaves challenged with RSI or exogenously-applied hormones were compared to those in the mock treatments at different time points, which were all set to ‘1’. Experiments were repeated three times with three independent biological repetitions each time. Error bars indicate the standard error. Asterisks indicate a significant difference (Fisher’s protected LSD test, * *p* < 0.05 or ** *p* < 0.01).

To examine the transcript levels of *CaSK23* in different pepper plant organs, total RNA from organs was isolated and used as templates for quantitative RT-PCR (qRT-PCR) with a specific primer pair of *CaSK23*, based on its 3′-UTR. The results demonstrated that *CaSK23* is constitutively expressed in all of the tested organs, including the root, stem, leaf, flower, and fruit. The highest level was found in the leaf, followed by the fruit (Figure 1). In addition, transcriptional expression of *CaSK23* in pepper leaves after RSI was also detected by qRT-PCR, the total RNA of pepper leaves inoculated with *R. solanacearum* strain FJC100301 [55], was isolated, and subjected to qRT-PCR. Inoculation of *R. solanacearum* significantly enhanced the relative transcriptional expression of a SA-dependent PR1 gene [56], but significantly down-regulated *CaSK23* after 3 hpi, indicating that CaSK23 might act as a negative regulator in pepper’s response to *R. solanacearum*. SA, JA, and ET act as general signaling molecules and play crucial roles in plant responses to pathogens. To assess the possible involvement of CaSK23 in signaling mediated by these hormones, the transcriptional abundance of *CaSK23* was determined by qRT-PCR in four-leaf pepper plants exogenously treated with SA, MeJA-, and ETH (ethephon, which can be converted to ethylene by plant metabolism). The validity of exogenous applications of tested hormones was confirmed by qRT-PCR analyses of the SA-, JA-, and ET-dependent marker genes such as *PR1* [56], *PIN2* [57], and *ACC oxidase* [57], respectively. The results revealed that the relative expression of *CaSK23* was reduced from 3 to 48 h post-treatment (hpt) in pepper leaves treated with 5 mM of SA or 100 μM MeJA. The lowest relative expression level of *CaSK23* in pepper leaves treated by the exogenous application of SA was observed from12 to 24 hpt, whereas that of the known SA-responsive marker gene *CaPR1* was dramatically up-regulated, while upon the application of MeJA, the lowest level of *CaSK23* transcript was observed at 12 hpt. The application of 10 mM of ETH did not affect the expression of *CaSK23* significantly (Figure 2).

### 2.3. Silencing of CaSK23 by VIGS Decreased Susceptibility of Pepper Plants to R. solanacearum

**Figure 3:**
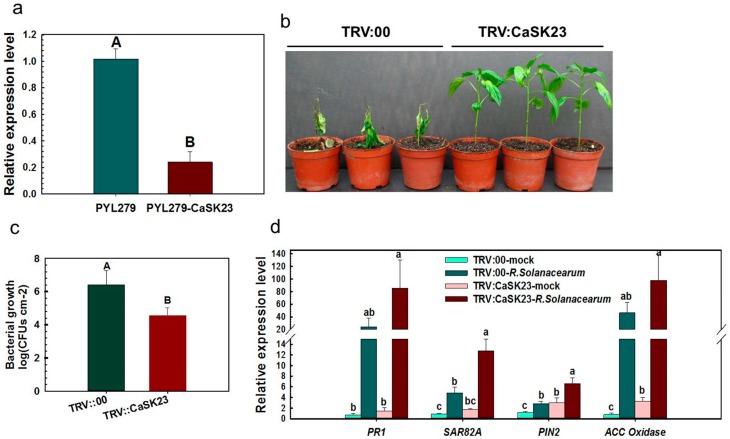
Virus-induced gene silencing of *CaSK23* decreased susceptibility of pepper to *R. solanacearum*. (**a**) The relative transcriptional expression of *CaSK23* in pYL279-*CaSK23* pepper plant inoculated with 5.0 mL of *R. solanacearum* suspension (OD_600_ = 0.8) by root irrigation at 72 h post inoculation (hpi), compared to that in pYL279 plants. (**b**) Disease symptoms of *CaSK23*-silenced and the empty vector control plants inoculated with *R. solanacearum* by root irrigation at 7 dpi. (**c**) *R. solanacearum* growth in the *R. solanacearum* inoculated third leaves of wild-type and CaSK23 silencing pepper plants at 2 dpi (days post inoculation); (**d**) *CaSK23* silencing significantly increased the expression of immunity-associated marker genes in pepper plants at 72 hpi with *R. solanacearum* by root irrigation, qne the transcripts of the marker genes were compared to that of mock treated TRV:00 plants, which were set to ‘1’. In (**a**,**c**,**d**), data represent the means ± SD from four biological replicates, and different letters above the bars indicate significant differences between means (uppercase letter *p* < 0.01; lowercase letter *p* < 0.05), determined by Fisher’s protected LSD test.

Since CaSK23 was found to be down-regulated by both RSI and exogenously-applied SA or MeJA, we speculated that CaSK23 might play a role in pepper immunity. To test this possibility, we employed a VIGS approach, which has been frequently used in functional genomic studies in *Solanaceae* [58,59,60,61]. To avoid the possible silencing of other homologues, a 139-bp specific fragment of *CaSK23* in the 3′-UTR was used to construct the VIGS vector pYL279-*CaSK23*. qRT-PCR was used to measure the silencing efficiency of *CaSK23* in *R. solanacearum*-inoculated *CaSK23*-VIGS pepper plants at 72 hpi. We found that the *CaSK23* transcript level was reduced to approximately 20% of that in pYL279 plants (Figure 3a). *CaSK23*-silenced pepper plants showed decreased disease symptoms compared to control plants at seven days post-inoculation (dpi), indicating that the silencing of CaSK23 attenuated *R. solanacearum* susceptibility (Figure 3b). In addition, the growth of *R. solanacearum* was found to be decreased in CaSK23 silencing plants compared to that in the control plants (Figure 3c). Consistent with decreased *R. solanacearum* susceptibility in CaSK23-silenced pepper plants, the transcript levels of defense-related pepper genes, including *CaPR1*, *CaSAR82A*, *CaPIN2*, and *ACC oxidase*, were all found to be enhanced in *CaSK23*-silenced plants compared to pYL279 pepper plants (Figure 3d).

### 2.4. Ectopic Overexpression of CaSK23 Increased Susceptibility of Transgenic Tobacco Plants to R. solanacearum 

**Figure 4:**
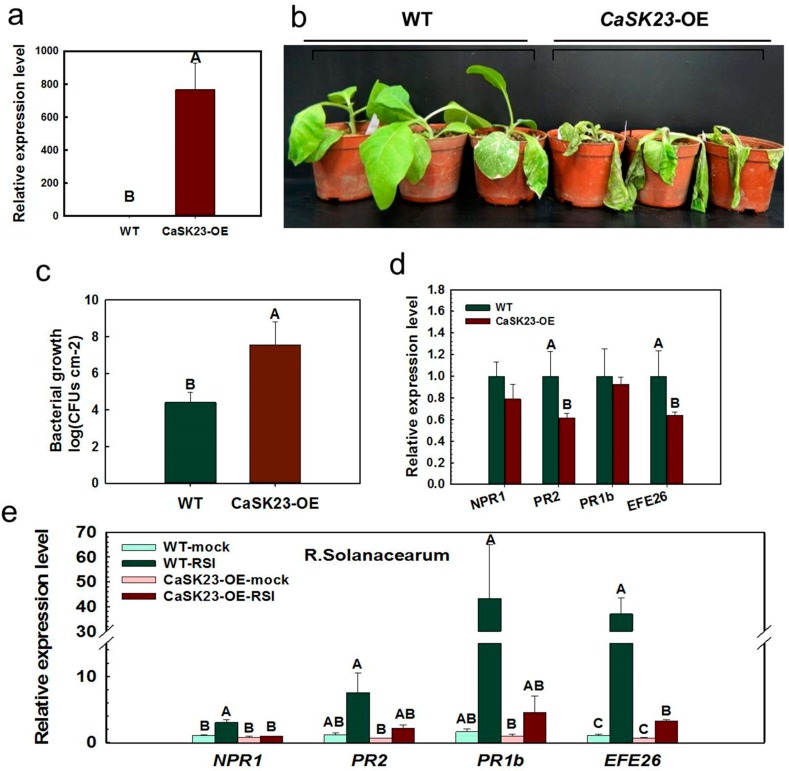
Transgenic tobacco plants overexpressing *CaSK23* displayed increased susceptibility to *R. solanacearum* inoculation. (**a**) The transcript abundance of CaSK23 in the transgenic tobacco plants, where NtEF-1α served as the endogenous control; (**b**) disease symptoms of plants of three eight-week-old transgenic tobacco lines and the wild-type K326 at 7 dpi inoculated with 5.0 mL of *R. solanacearum* suspension (OD_600_ = 0.8) by root irrigation; (**c**) *R. solanacearum* growth in third leaves of wild-type and CaSK23 overexpressing tobacco plants at 48 hpi (hours post inoculation); (**d**) relative transcript levels of defense-related genes (*NPR1*, *PR2*, *PR1b*, and *EFE26*) at indicated time points in transgenic tobacco plants overexpressing *CaSK23* without RSI compared to that in the wild-type plants, which were all set to ‘1’; (**e**) relative transcript levels of defense-related genes in *CaSK23*-overexpressing transgenic tobacco and wild-type plants at 72 h post inoculation with *R. solanacearum,* compared to that in mock treated wild-type K326 plants, which were all set to ‘1’. In a, c, d, and e, data represent the means ± SD from four biological replicates, and different uppercase letters above the bars indicate significant differences between means (*p* < 0.01), determined by Fisher’s protected LSD test.

Because it is difficult to obtain transgenic pepper plants, we generated transgenic tobacco lines and used the T_3_ homozygous plants, which constitutively express *CaSK23*, for the functional characterization of CaSK23 in plant immunity. No phenotypic differences were found among plants of the nine T_3_ lines and the K326 tobacco plants, while high transcript levels of *CaSK23* were detected in the T_3_ lines (Figure 4a). The FJC100301 strain of *R. solanacearum* was used to inoculate the plants of five randomly selected T_3_ lines and K326 plants. All tested transgenic lines exhibited enhanced disease symptoms in response to RSI at 7 dpi compared to K326 plants. One line (CaSK23-OE1) was selected to assay in detail, and clear wilting symptoms were observed in CaSK23-OE1 plants inoculated with FJC100301 at 7 dpi, whereas K326 plants exhibited only slight wilting symptoms (Figure 4b). In addition, the growth of *R. solanacearum* was found to be enhanced by the overexpression of CaSK23, since a significantly higher number of colony-forming units (cfu) of the pathogen at 48 hpi were found in *R. solanacearum* inoculated CaSK23-OE1 plants compared to the wild-type tobacco plants (Figure 4c).

In addition, we measured the transcription of immunity-associated genes, including SA-responsive genes *NtNPR1* and *NtPR2*, JA-responsive *NtPR1b*, and ET-biosynthesis-associated *NtEFE26*, in transgenic tobacco plants without RSI. We found that expression levels of tested genes in transgenic tobacco plants were similar to or below those in K326 plants (Figure 4d). Although immunity-associated genes were up-regulated by RSI to varying degrees, the tested immunity-associated marker genes were significantly lower in transgenic tobacco plants than those in wild-type (WT) plants (Figure 4e).

### 2.5. Transient Expression of CaSK23 Suppressed Expression of Defense-Associated Marker Genes in Pepper Leaves

**Figure 5:**
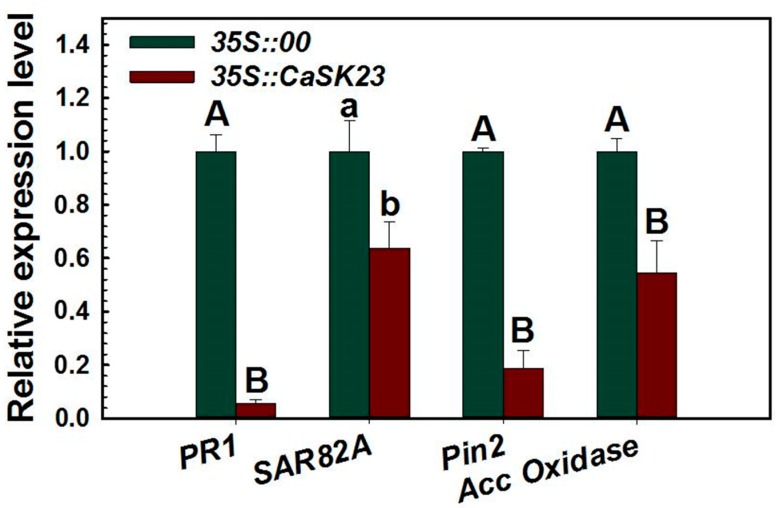
qRT-PCR analysis of relative expression levels of immunity-associated genes in pepper plants transiently overexpressing *CaSK23*. Pepper leaves were infiltrated with *agrobacterium* GV3101 cells carrying the *35S::00* (empty vector) or *35S::CaSK23* construct, and total leaf RNA was isolated at 24 hpi for qRT-PCR analysis. Relative transcript levels of the marker genes in *35S::CaSK23* expressing pepper leaves were compared to those in the *35S::00* control plants, which were all set to ‘1’. Different letters indicate significant differences (*p* < 0.01) from three independent biological replicates based on Fisher’s protected LSD test.

The transient expression approach, which has been frequently utilized to analyze the potential role of a given gene in immunity in *Solanaceae* [62,63], was employed to confirm the results from tobacco plants, in which the ectopic overexpression of *CaSK23* compromised immunity. This result demonstrated that transient overexpression of *CaSK23* significantly down-regulated all of the tested immunity-associated marker genes, including *CaPR1*, *CaSAR82A*, *CaPIN2*, and *ACC oxidase* (Figure 5), which were found to be enhanced in *R. solanacearum*-inoculated *CaSK23*-silenced pepper plants.

## 3. Discussion

While the roles of GSK3 kinases are well established in BR signaling for plant growth and development, limited information exists about the roles of GSK3 kinases in plant immunity, especially in non-model plants like peppers. In the present study, our data indicated that a putative GSK3, termed CaSK23, acts as a negative regulator in the pepper response to RSI.

The evidence that *CaSK23* acts as a negative regulator in pepper response to *R. solanacearum* attack comes from the following data. *CaSK23* silencing by VIGS in pepper plants significantly boosted the resistance of pepper plants to RSI, which was confirmed by the accompanied upregulation of PR genes, including *CaPR1*, *CaBPR1*, *CaSAR82A*, *CaPIN2*, and *ACC oxidase* in *CaSK23*-VIGS pepper plants. These genes were previously found to confer pepper disease resistance and have been frequently used as immunity-associated SA-, JA-, or ET-dependent marker genes [57,64,65,66,67,68,69]. In contrast, the overexpression of *CaSK23* significantly compromised the resistance of transgenic tobacco plants to RSI, and the PR genes that have been previously linked to disease resistance in tobacco, such as SA-responsive *NtNPR1* [70,71], *NtPR1* [71], JA-responsive *NtPR1b* [72], and ET-biosynthesis-related *EFE26* [72], were down-regulated or unchanged in *CaSK23*-overexpressing plants that were not inoculated with *R. solanacearum*, compared to WT plants. However, in *CaSK23*-overexpressing plants that were inoculated with *R. solanacearum*, all tested PR genes were significantly down-regulated compared to WT control plants, suggesting that transcriptional modification of these PR genes by overexpression of *CaSK23* was influenced by *R. solanacearum* attack. A similar phenomenon of transcriptional modification of target genes by *R. solanacearum* was observed in our previous functional characterization of *CaWRKY27* and *CaWRKY40* [55,73]. Consistently, in pepper leaves transiently overexpressing *CaSK23*, PR genes were also significantly down-regulated compared to control leaves. All these data strongly suggest that *CaSK23* acts as a negative regulator in pepper’s response to *R. solanacearum* attack. As plant immunity is an energy- and resource-intensive process [74], it should be tightly regulated. Negative regulators have been found to be frequently involved in the regulation of plant immunity [3,60,75]; these regulators are thought to prevent the appropriate activation of defense responses at suboptimal concentrations of signal molecules or to turn off systemic acquired resistance (SAR) once the invasion of pathogens has been resolved [75]. Since *CaSK23* is constitutively expressed in pepper plants and is down-regulated by RSI, we speculate that this expression in the absence of pathogen attack may play a role in attenuating immunity to reduce fitness costs, and this restrained immunity may be released by down-regulation of *CaSK23* when the pepper plants are challenged by pathogens such as *R. solanacearum*.

The plant hormones SA, JA, and ET play key roles in the regulation of the defense signaling network that is recruited upon perception of an invader [76,77,78,79]. SA-induced defenses were originally found to be specific in a plant’s response to pathogens with a biotrophic lifestyle, whereas JA- or ET-mediated defense is involved in necrotrophic pathogens [78]. The signaling pathways mediated by SA and JA have been found to interact with each other, and it is believed that they interact synergistically in PAMP triggered immunity, but in a compensatory manner in effector triggered immunity [80,81]. Our present data showed that the transcriptional expression of *CaSK23* was significantly down-regulated by the exogenous application of SA or MeJA, but not by that of ETH. As synergistic relationships between SA, JA, and ET signaling have been previously found in pepper immunity against *R. solanacearum* [55,58,59,82], the down-regulation of *CaSK23* by *R. solanacearum* inoculation was consistent with its down-regulation by the exogenous application of SA or MeJA. This result further supports the role of *CaSK23* as a negative regulator in pepper immunity against *R. solanacearum.* However, inconsistent to that, *CaSK23* was not down-regulated by the exogenous application of ETH, and ET signaling associated marker gene ACC oxidase or EFE26 was upregulated in *CaSK23*-VIGS pepper plants and down-regulated in *CaSK23* overexpressing tobacco plants or *CaSK23* transiently overexpressing pepper plants. One explanation for this inconsistency is that some of the possible convergent components of SA-, JA-, and ET-dependent signaling might be modified by CaSK23. For example, CabZIP63, CaWRKY27, CaWRKY40, and CaWRKY58 were previously found to be synergistically regulated by exogenous SA, JA, or ET, and they can synergistically modify SA-, JA-, and ET-dependent signaling pathways [55,60,73,82,83]. Additionally, CaSK23 might synergistically modify the SA-, JA-, and ET-dependent signaling through modification of these kind of convergent components. Identification of the possible downstream convergent components in the future would provide insight into the underlying mechanism of immunity mediated by CaSK23.

**Figure 6:**
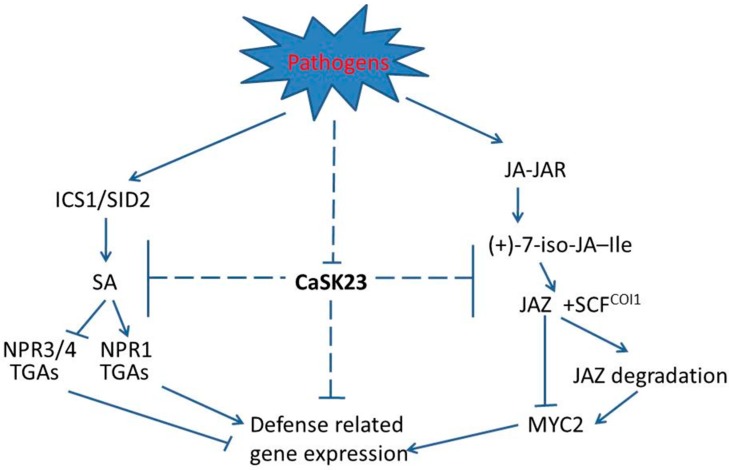
Hypothetical model for the role of CaSK23 in pepper response to *R. solanacearum* infection. Upon the challenge of *R. solanacearum* infection, *CaSK23* is transcriptionally downregulated, as CaSK23 acts as a negative regulator in pepper immunity partially by blocking SA- and JA-dependent signaling, and the downregulation of CaSK23 releases SA and JA dependent signaling, leading to enhanced immunity against *R. solanacearum* infection. In SA signaling, the production of SA triggered by pathogens is sensed and bound by receptors such as NPR1 and NPR3/NPR4, in which NPR1 acts positively in plant immunity by interacting with TGAs, while NPR3/NPR4 act negatively by interacting with TGAs in SA dependent immunity [85]. In non-stress challenged plants, immune signaling mediated by the MYC transcription factor is depressed via its interaction with JAZs. When plants are attacked by a pathogen or herbivore, JA is produced, which conjugates with amino acids by JAR1, resulting in enhanced (+)-7-iso-JA–Ile levels. The increased (+)-7-iso-JA–Ile favours the formation of JAZ–COI1 complexes, leading to the ubiquitination and degradation of JAZ by the 26S proteasome, thus MYC2 is released to activate transcription [86]. T-bar mean repression. Solid lines represent clear pathways, but dotted lines are that not clear.

Taken together, our data suggest that CaSK23 acts as a negative regulator in pepper’s response to *R. solanacearum* attack, and the constitutive expression of CaSK23 might partially suppress immunity by blocking SA and JA dependent signaling in the absence of pathogen, while its decreased expression derepresses immunity when pepper plants are attacked by pathogens (Figure 6). As GSK3 kinases have been implicated in BR signaling [33,34,35,36,37,38], crosstalk between SA-, JA-, and BR-dependent signaling has frequently been found [22,84], and the possible role of CaSK23 in BR signaling and in crosstalk between SA-, JA-, and BR-mediated signaling remains to be elucidated in the future.

## 4. Materials and Methods

### 4.1. Plant Materials and Growth Conditions

The seeds of pepper (*Capsicum annuum*) inbred line 8^#^, tobacco (*Nicotianatabacum*) cultivar K326, and its T_2_ or T_3_ transgenic lines, were sown in a soil mix [peat moss:perlite, 2:1 (*v*/*v*)] in plastic pots. Pots were placed in a growth room under conditions of 25 °C, 60–70 μmol photons m^−2^·s^−1^, a relative humidity of 70%, and a 16/8 h photoperiod.

### 4.2. Isolation of CaSK23 and Its Sequence Analysis

A cDNA-AFLP profiling experiment searching for genes that differentially respond to exogenous SA application identified a transcript-derived fragment (TDF) that was down-regulated by exogenous SA treatment in pepper plants (our unpublished data), with a high sequence similarity to associated GSK3-like kinases. Full-length cDNAs were isolated by PCR-based 96-well screening with the specific primers derived from TDFs described by Munroe and colleagues [87]. Positive clones (λTriplEx2) were converted to a pTriplEx2 by in vivo excision following the user manual and sequenced by TaKaRa (Dalian, China).

### 4.3. Vector Construction

To construct a vector for overexpression, the full-length open reading frame (ORF) of *CaSK23* was cloned into the entry vector pDONR207 by a BP reaction, and was then transferred into the destination vector pK7WG2 (*35S::CaSK23*) by an LR reaction (Invitrogen, Carlsbad, CA, USA). To construct a vector for virus-induced gene silencing (VIGS), a part of the 3′-Untranslated Region (UTR) of *CaSK23* was cloned into the destination vector pYL279 by the same gateway technology (Invitrogen, Carlsbad, CA, USA). All the primers used in vector construction are listed in Table S1.

### 4.4. Pathogens and Inoculation Procedures

A highly virulent *R. solanacearum strain* FJC100301 was isolated from wilted pepper samples from the Fujian province (China) and amplified according to a previously described method [55].

For total RNA isolation and real-time RT-PCR analysis of the expression of *CaSK23* against RSI, pepper plants were inoculated by injecting 10 µL of the resulting *R. solanacearum* suspension (OD_600_ = 0.8) into the fourth leaves from the top using a syringe with a needle at its sixth to eighth leaf stage. The respective third leaves were harvested at the indicated time points for the preparation of RNA. For the phenotypic effect analysis of *CaSK23* overexpression and *CaSK23* silencing on RSI and on the expression of immunity associated maker genes in tobacco plants and *CaSK23-VIGS* pepper plants, respectively, the root of tobacco or pepper plants were slightly wounded by a pair of sterile scissors, and the wounded plants were inoculated with 5.0 mL *R. solanacearum* suspension (OD_600_ = 0.8) by root irrigation and were harvested at indicated time points.

### 4.5. Treatment of Plants with Exogenous Hormones

Pepper plants at the four-leaf stage were sprayed with 5 mM salicylic acid (SA) (in 10% distilled ethanol), 100 µM methyl jasmonate (MeJA) (in 10% distilled ethanol), or 10 mM ethephon (ETH) (in sterile ddH_2_O). Mock treatments were performed by spraying with corresponding solvents or ddH_2_O.

### 4.6. VIGS of CaSK23 in Pepper Plants 

For VIGS of *CaSK23* in pepper plants, GV3101 cells containing pYL192, in addition to GV3101 cells containing pYL279-*CaSK23* or pYL279 (negative control), were resuspended in the induction medium (10 mM MES, 10 mM MgCl_2_, 200 µM acetosyringone, pH 5.6), respectively, and mixed at a 1:1 ratio. The mixed GV3101 cells were co-injected into the cotyledons of two-week-old pepper plants. Our previous studies report more details on this process [55,58,59,73,82].

### 4.7. Transient Expression of CaSK23 in Pepper Leaves

For transient expression analysis, GV3101 harboring either *35S::CaSK23* or *35S::00* (negative control) was grown overnight, separately, and then resuspended in induction medium. The bacterial suspension (OD_600_ = 0.8) was injected into leaves of pepper plants at the eight-leaf stage using a syringe without a needle, and then injected leaves were collected at indicated time points for further analysis.

### 4.8. Construction of Transgenic CaSK23-Overexpressing Tobacco Lines

Leaf disks of tobacco (*N. tabacum* cv.) K326 were transformed with GV3101 harboring *35S::CaSK23*, as described previously [88]. The initial transgenic tobacco lines (T_0_) were selected by kanamycin, and seven transgenic lines were further confirmed by *CaSK23*-specific primers by PCR and RT-PCR, respectively. T_1_ seeds were collected from regenerated T_0_ plants, T_2_ or T_3_ seeds were collected in a similar way, and T_3_ seeds were used for future analysis.

### 4.9. Quantitative Real-Time RT-PCR

To determine the relative transcription levels of selected genes, qPCR was performed with specific primers (Table S1) according to the manufacturer’s instructions for the BIO-RAD Real-time PCR system (Foster City, CA, USA) and the SYBR Premix Ex Taq II system (TaKaRa). The procedures for total RNA preparation and qPCR were carried out as previously reported [73,82,89]. Three independent biological replicates of each treatment were performed, and three technical replicates were performed for each biological replicate. Data were analyzed using the Livak method [90]. Relative transcript levels of analyzed pepper or tobacco genes were normalized to the transcript levels of CaActin or NtEF1α, respectively. All primers used in qPCRare are listed in Table S1.

### 4.10. Accession Numbers

Sequence data from this article can be found in the GenBank/EMBL data libraries under the following accession numbers: pepper *CaPR1* (AF053343), pepper *CaSAR82A* (AF313766), pepper *CaPIN2* (AAB94771), pepper *CaACC Oxidase* (AB434925), pepper *CaActin* (AY572427), tobacco *NtNPR1* (U76707), tobacco *NtPR2* (M60460), tobacco *NtPR1b* (X66942), tobacco *NtEFE26* (Z29529), and tobacco *NtEF1a* (D63396).

## Supplementary Materials

Supplementary materials can be found at http://www.mdpi.com/1422-0067/19/9/2698/s1.
