# Supplementary material for: CaSK23, a Putative GSK3/SHAGGY-Like Kinase of Capsicum annuum, Acts as a Negative Regulator of Pepper’s Response to Ralstonia solanacearum Attack
Source: Int J Mol Sci. 2018 Sep 11;19(9):2698. doi: 10.3390/ijms19092698 (PMC6163794; doi:10.3390/ijms19092698)
Supplement: Supplementary file 1 [file ijms-19-02698-s001.pdf]

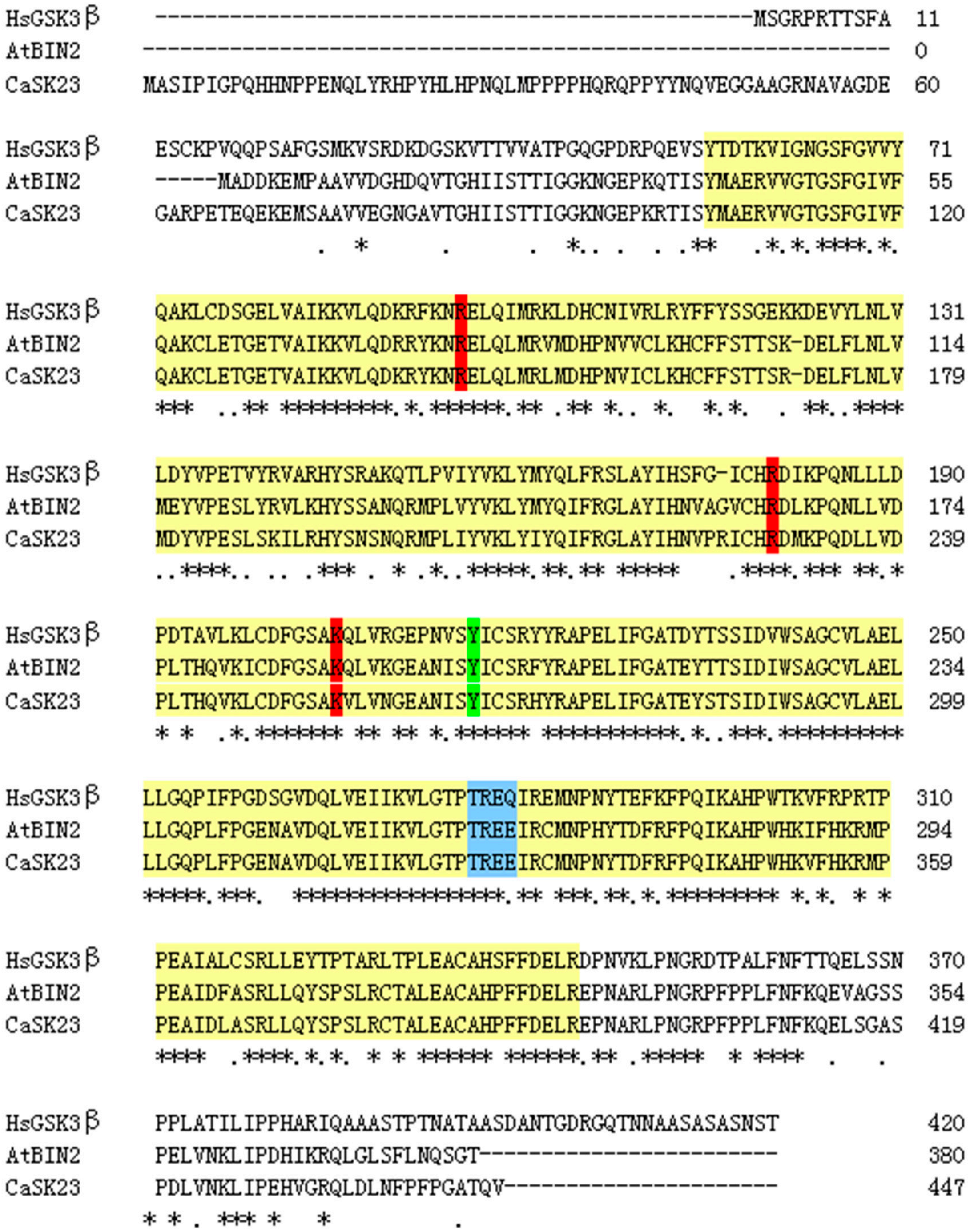
HsGSK3β ---MSGRPRTTSFA 11
AtBIN2 -- 0
CaSK23 MASIPIGPQHHNPPENQLYRHPYHLHPNQLMPPPPHQRQPPYYNQVEGGAAGRNAVAGDE 60
HsGSK3β ESCKPVQQPSAFGSMKVSRDKDGSKVTTVVATPGQGPDRPQEVSYTDTKVIGNGSFGVVY 71
AtBIN2 -----MADDKEMPAAVVDGHDQVTGHIISTTIGGKNGEPKQTISYMAERVVGTGSFGIVF 55
CaSK23 GARPETEQEKEMSAAVVEGNGAVTGHIISTTIGGKNGEPKRTISYMAERVVGTGSFGIVF 120
HsGSK3β QAKLCDSGELVAIKKVLQDKRFKNRELQIMRKLDHCNIVRLRYFFYSSGEKKDEVYLNLV 131
AtBIN2 QAKCLETGETVAIKKVLQDRRYKNRELQLMRVMDHPNVVCLKHCFFSTTSK-DELFLNLV 114
CaSK23 QAKCLETGETVAIKKVLQDKRYKNRELQLMRLMDHPNVICLKHCFFSTTSR-DELFLNLV 179
HsGSK3β LDYVPETVYRVARHYSRAKQTLPVIYVKLYMYQLFRSLAYIHSFG-ICHRDIKPQNLLLD 190
AtBIN2 MEYVPESLYRVLKHYSSANQRMPLVYVKLYMYQIFRGLAYIHNVAGVCHRDLKPQNLLVD 174
CaSK23 MDYVPESLSKILRHYSNSNQRMPLIYVKLYIYQIFRGLAYIHNVPRICHRDMKPQDLLVD 239
HsGSK3β PDTAVLKLCDFGSAKQLVRGEPNVSYICSRYYRAPELIFGATDYTSSIDVWSAGCVLAEL 250
AtBIN2 PLTHQVKICDFGSAKQLVKGEANISYICSRFYRAPELIFGATEYTTSIDIWSAGCVLAEL 234
CaSK23 PLTHQVKLCDFGSAKVLVNGEANISYICSRHYRAPELIFGATEYSTSIDIWSAGCVLAEL 299
HsGSK3β LLGQPIFPGDSGVDQLVEIIKVLGTPTREQIREMNPNYTEFKFPQIKAHPWTKVFRPRTP 310
AtBIN2 LLGQPLFPGENAVDQLVEIIKVLGTPTREEIRCMNPHYTDFRFPQIKAHPWHKIFHKRMP 294
CaSK23 LLGQPLFPGENAVDQLVEIIKVLGTPTREEIRCMNPNYTDFRFPQIKAHPWHKVFHKRMP 359
HsGSK3β PEAIALCSRLLEYTPTARLTPLEACAHSFFDELRDPNVKLPNGRDTPALFNFTTQELSSN 370
AtBIN2 PEAIDFASRLLQYSPSLRCTALEACAHPFFDELREPNARLPNGRPFPPLFNFKQEVAGSS 354
CaSK23 PEAIDLASRLLQYSPSLRCTALEACAHPFFDELREPNARLPNGRPFPPLFNFKQELSGAS 419
HsGSK3β PPLATILIPPHARIQAAASTPTNATAASDANTGDRGQTNNAASASASNST 420
AtBIN2 PELVNKLIPDHIKRQLGLSFLNQSGT------------------------ 380
CaSK23 PDLVNKLIPEHVGRQLDLNFPFPGATQV---------------------- 447

(**a**)

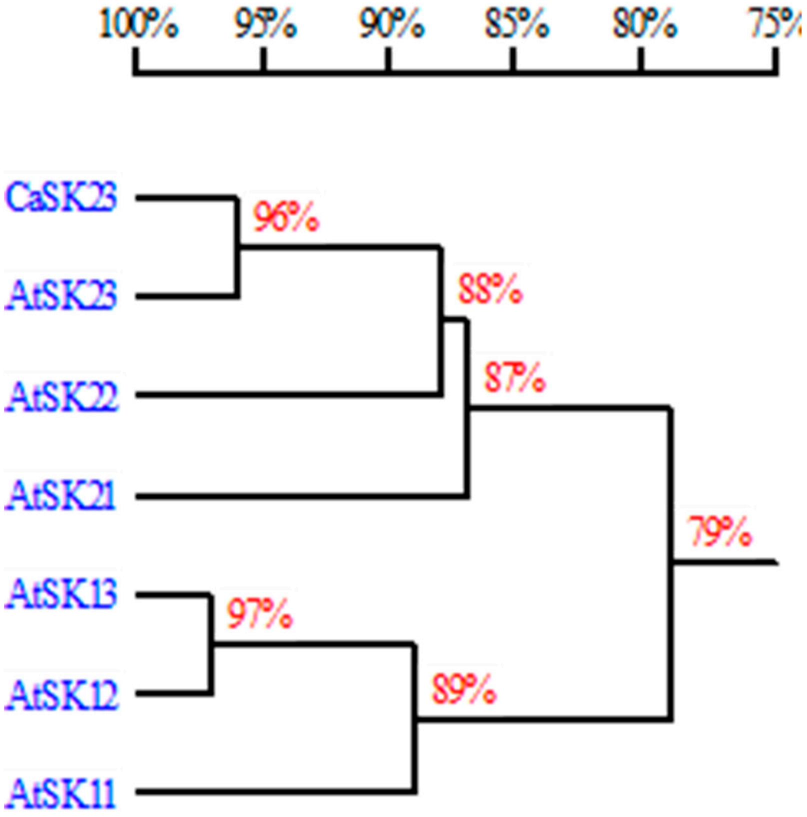

(**b**)

Figure S1. (**a**) Comparison of amino acid sequences of CaSK23 with *Homo sapiens* GSK3β (HsGSK3β, NP_001139628) and *Arabidopsis thaliana* BIN2 (AtBIN2, At4G18710) with DNAMAN5.0. The kinase domain is highlighted in yellow, and the conserved tyrosine (Y265 in CaGSK23) whose phosphorylation is required for kinase activity is highlighted in green. The conserved phosphate-binding residues (R144, R229, and K254 in CaSK23) that interact with prime-phosphorylated substrates are highlighted in red. The conserved TREE domain is in cyan. The figure was made based on the figure of Saidi et al [54]. (**b**) Phylogenetic tree analysis was made by DNAMAN5.0 with amino acid sequences of CaSK23 and its homologues in Arabidopsis including At5G26751（AtSK11）, At3G05840（AtSK12）, At5G14640（AtSK13）, At4G18710（AtSK21/AtBIN2）, At1G06390（AtSK22）, and At2G30980（AtSK23).

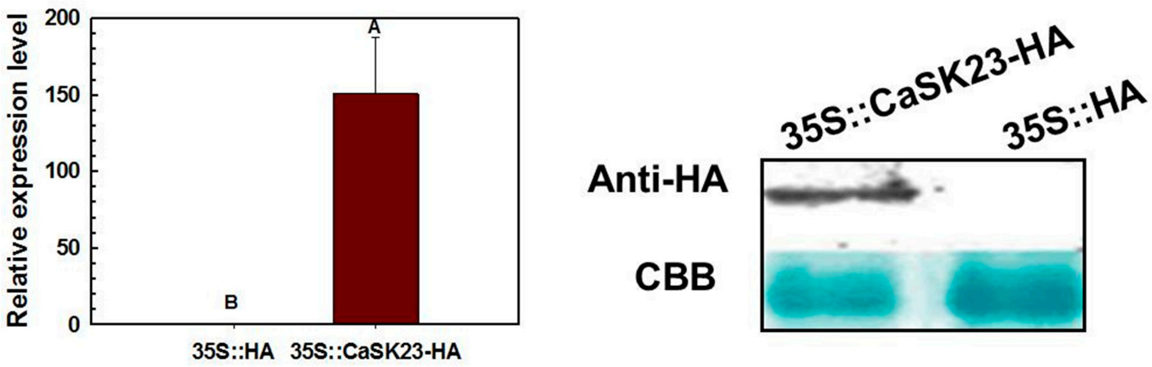

Figure S2. Expression of *CaSK23-HA* in pepper leaves via agroinfiltration. a. The transcript levels of *CaSK23* in agro-infiltrated pepper leaves with GV3101 cells containing *35S::CaSK23-HA* or 35S::*HA* by qRT-PCR with a specific primer pair of *CaSK23* at 24hpi. Data represent the means ± SD obtained from five independent experiments. Different

letters above the bars indicate significant differences among means ($p < 0.01$), determined by Fisher's protected LSD test. b. The expression of CaSK23 in agro-infiltrated pepper leaves with GV3101 cells containing *35S:: CaSK23-HA* by western blotting, and the total proteins of *CaSK23-HA* transiently overexpressing pepper leaves were isolated at 24 hpi and were immunoblotted with antibodies of HA.

Table S1. Primers for PCR and qRT-PCR used in this study.

Gene	Accession No.	Forward primers (5'→3')	Reverse primers(5'→3')
***Primers used for construction of over-expression & subcellular localization vectors**			
CaSK23		AAAAAGCAGGCTACATGGCTTCTA TACCCATTGGT	AGAAAGCTGGGTCCTACACTTGTG TTGCACCGG
***Primers used for construction of VIGS vector**			
CaSK23		AAAAAGCAGGCTACCAGAAAAT ATAGGGAGTCCTTG	AGAAAGCTGGGTCCATAAGTAGA CAGGATTACAC
***Primers used for qRT-PCR analyses in pepper plants**			
CaSK23		TGGCAGAGCGAGTTGTCGGTAC	TGAGCGGCATCCTTTGATTTGA
CaPR1	AF053343	GCCGTGAAGATGTGGGTCAATGA	TGAGTTACGCCAGACTACCTGAGT A
CaSAR82A	AF313766	CCATAGAGGT TCATATGGAA GTCC	GGAAAGTGAA TAGGACCTTT
CaPIN2	AAB94771	GCAACTATTACAGCGTCATCGG	GGGTCAGACTCTCCTTCACAAA
CaACC Oxidase	AB434925	CCATTGTGGTCAACCTTGGC	GCATCGCTTCCTGGATTGTAA
CaActin	AY572427	AGGGATGGGTCAAAAGGATGC	GAGACAACACCGCCTGAATAGC
***Primers used for qRT-PCR analyses in tobacco plants**			
NtNPR1	U76707	GGCGAGGAGTCCGTTCTTTAA	TCAACCAGGAATGCCACAGC
NtPR2	M60460	TGATGCCCTTTTGGATTCTATG	AGTTCCTGCCCCGCTTT
NtPR1b	X66942	AACCCATCCATACTATTCCTTG	GCCGCTAACCTATTGTCCC
NtEFE26	Z29529	CGGACGCTGGTGGCATAAT	CAACAAGAGCTGGTGCTGGATA
NtEF1a	D63396	TGCTGCTGTAACAAGATGGATGC	GAGATGGGGACAAAGGGGATT

* The utlizations of the primes were listed in the lines with gray background.
